# Active Monitoring of Persons Exposed to Patients with Confirmed COVID-19 — United States, January–February 2020

**DOI:** 10.15585/mmwr.mm6909e1

**Published:** 2020-03-06

**Authors:** Rachel M. Burke, Claire M. Midgley, Alissa Dratch, Marty Fenstersheib, Thomas Haupt, Michelle Holshue, Isaac Ghinai, M. Claire Jarashow, Jennifer Lo, Tristan D. McPherson, Sara Rudman, Sarah Scott, Aron J. Hall, Alicia M. Fry, Melissa A. Rolfes

**Affiliations:** ^1^COVID-19 Response Team, CDC; ^2^Orange County Health Care Agency, California; ^3^San Benito County Public Health Services, California; ^4^Wisconsin Department of Health Services; ^5^Washington Department of Health; ^6^Epidemic Intelligence Service, CDC; ^7^Illinois Department of Public Health; ^8^Los Angeles County Department of Public Health, California; ^9^Boston Public Health Commission, Massachusetts; ^10^Chicago Department of Public Health, Illinois; ^11^County of Santa Clara Public Health Department, California; ^12^Maricopa County Department of Public Health, Arizona.

In December 2019, an outbreak of coronavirus disease 2019 (COVID-19), caused by the virus SARS-CoV-2, began in Wuhan, China (*1*). The disease spread widely in China, and, as of February 26, 2020, COVID-19 cases had been identified in 36 other countries and territories, including the United States. Person-to-person transmission has been widely documented, and a limited number of countries have reported sustained person-to-person spread.* On January 20, state and local health departments in the United States, in collaboration with teams deployed from CDC, began identifying and monitoring all persons considered to have had close contact^†^ with patients with confirmed COVID-19 (*2*). The aims of these efforts were to ensure rapid evaluation and care of patients, limit further transmission, and better understand risk factors for transmission.

As of February 26, 12 travel-related COVID-19 cases had been diagnosed in the United States, in addition to three COVID-19 cases in patients with no travel history (including two cases in close household contacts) and 46 cases reported among repatriated U.S. citizens.^§^ Following confirmed diagnosis, the 12 patients with travel-related COVID-19 were isolated in the hospital if medically necessary, or at home once home care was deemed clinically sufficient.^¶^ Among the first 10 patients with travel-related confirmed COVID-19 reported in the United States, a total of 445 persons (range = 1–201 persons per case) who had close contact with one of the 10 patients on or after the date of the patient’s symptom onset were identified. Nineteen (4%) of the 445 contacts were members of a patient’s household, and five of these 19 contacts continued to have household exposure to the patient with confirmed COVID-19 during the patient’s isolation period; 104 (23%) were community members who spent at least 10 minutes within 6 feet of a patient with confirmed disease; 100 (22%) were community members who were exposed** to a patient in a health care setting; and 222 (50%) were health care personnel.^††^

Active symptom monitoring of the 445 close contacts, consisting of daily telephone, text, or in-person inquiries about fever or other symptoms for 14 days following the last known exposure to a person with confirmed COVID-19, was conducted by local health jurisdictions. During the 14 days of active symptom monitoring, 54 (12%) close contacts developed new or worsening symptoms deemed by local public health authorities to be concerning for COVID-19 and were thus considered persons under investigation (PUIs)^§§^ and subsequently were tested for SARS-CoV-2. Two persons who were household members of patients with confirmed COVID-19 tested positive for SARS-CoV-2. This yielded a symptomatic secondary attack rate of 0.45% (95% confidence interval [CI] = 0.12%–1.6%) among all close contacts,^¶¶^ and a symptomatic secondary attack rate of 10.5% (95% CI = 2.9%–31.4%) among household members. Both persons with confirmed secondary transmission had close contact with the respective source patient before COVID-19 was confirmed and were isolated from the source patient after the patient’s COVID-19 diagnosis.

No other close contacts who were tested for SARS-CoV-2 had a positive test, including the five household members who were continuously exposed during the period of isolation of their household member with confirmed COVID-19. An additional 146 persons exposed to the two patients with secondary COVID-19 transmission underwent 14 days of active monitoring. Among these, 18 (12%) developed symptoms compatible with COVID-19 and were considered PUIs. All tested negative, and no further symptomatic COVID-19 cases (representing tertiary transmission) have been identified.

In the United States, two instances of person-to-person transmission of SARS-CoV-2 have been documented from persons with travel-related COVID-19 to their household contacts. Since February 28, an increasing number of newly diagnosed confirmed and presumptive COVID-19 cases have been in patients with neither a relevant travel history nor clear epidemiologic links to other confirmed COVID-19 patients. However, despite intensive follow-up, no sustained person-to-person transmission of symptomatic SARS-CoV-2 was observed in the United States among the close contacts of the first 10 persons with diagnosed travel-related COVID-19. Analyses of timing of exposure during each patient’s illness as well as the type and duration of exposures will provide information on potential risk factors for transmission. Infection control and prevention efforts by patients with COVID-19, their household members, and their health care providers,*** in combination with contact tracing activities, are important to mitigate community spread of the disease.

## Footnotes

* https://www.cdc.gov/coronavirus/2019-ncov/travelers/index.html.

† Close contact was defined by the state and local health jurisdictions with reference to the following online guidance: https://www.cdc.gov/coronavirus/2019-ncov/hcp/guidance-risk-assesment-hcp.html.

§ https://emergency.cdc.gov/han/2020/han00428.asp.

¶ https://www.cdc.gov/coronavirus/2019-ncov/hcp/guidance-prevent-spread.html.

** For these investigations, exposure of community members within a health care setting was defined as either at least 10 minutes spent within 6 feet of the patient with confirmed COVID-19 (e.g., in a waiting room) or having spent time in the same airspace (e.g., the same examination room) for 0–2 hours after the confirmed COVID-19 patient. The duration of time in the same airspace after the patient with confirmed COVID-19 was applied differently by health jurisdictions. However, no contacts were enumerated among those who were in the same airspace >2 hours after the patient with confirmed COVID-19.

†† Health care personnel were defined as volunteers or paid persons who serve in a health care setting who might come into direct or indirect contact with patients or infectious materials. Examples of close contact with a patient or with infectious material could include spending prolonged time within 6 feet of the patient, conducting or being present during an aerosol-generating procedure, or direct contact with the patient’s secretions or excretions. Interim guidance for assessing the exposure risk and for symptoms that should prompt further evaluation among health care personnel is available at https://www.cdc.gov/coronavirus/2019-ncov/hcp/guidance-risk-assesment-hcp.html.

§§ At the time of the investigations, persons with close contact to a confirmed COVID-19 patient could be considered PUIs if they developed fever or signs or symptoms of lower respiratory tract illness. This threshold might be lower for contacts who are health care workers. At this time, symptomatic close contacts of a patient with confirmed COVID-19 should be further evaluated in consultation with public health authorities to review signs or symptoms and possible exposure on a case-by-case basis. Further information is available at https://www.cdc.gov/coronavirus/2019-nCoV/hcp/clinical-criteria.html.

¶¶ The 95% confidence interval around the binomial proportion was calculated using the Wilson score interval.

*** https://www.cdc.gov/coronavirus/2019-ncov/hcp/guidance-prevent-spread.html; https://www.cdc.gov/coronavirus/2019-ncov/infection-control/control-recommendations.html.
